# Vav1 Down-Modulates Akt2 Expression in Cells from Pancreatic Ductal Adenocarcinoma: Nuclear Vav1 as a Potential Regulator of Akt Related Malignancy in Pancreatic Cancer

**DOI:** 10.3390/biomedicines8100379

**Published:** 2020-09-26

**Authors:** Silvia Grassilli, Federica Brugnoli, Rossano Lattanzio, Simonetta Buglioni, Valeria Bertagnolo

**Affiliations:** 1 Department of Morphology, Surgery and Experimental Medicine, University of Ferrara, 44121 Ferrara, Italy; silvia.grassilli@unife.it (S.G.); bgf@unife.it (F.B.); 2 LTTA Centre, University of Ferrara, 44121 Ferrara, Italy; 3 Department of Medical, Oral and Biotechnological Sciences, Center for Advanced Studies and Technology (CAST), ‘G. d’Annunzio’ University of Chieti-Pescara, 66100 Chieti, Italy; rossano.lattanzio@unich.it; 4 Pathology Unit, IRCCS Regina Elena National Cancer Institute, 00144 Rome, Italy; simonetta.buglioni@ifo.gov.it

**Keywords:** pancreatic ductal adenocarcinoma, Vav1, targeting Akt pathways

## Abstract

Pancreatic ductal adenocarcinoma (PDAC) is the most aggressive tumor malignancy worldwide, mainly due to uncontrolled metastasis. Among the numerous molecules deregulated in PDAC, different members of the Akt pathways are of great importance because they are involved in tumor cell proliferation, migration, and invasion. We have recently demonstrated that Vav1, ectopically expressed in solid tumors, is capable of down-modulating expression and/or activation of specific Akt isoforms in breast cancer cells. By using pancreatic cell lines expressing different basal levels of Vav1, we demonstrated here that Vav1 down-regulates the expression of Akt2, known to correlate with tumor metastases and resistance to therapy. In particular, while the silencing of Vav1 is sufficient to induce Akt2, its up-modulation reduces Akt2 levels only when Vav1 accumulates inside the nucleus of PDAC cells. Moreover, in PDAC tissues, we revealed that high nuclear levels of Vav1 correlate with low Akt2 expression. Although we cannot demonstrate the mechanisms involved, our results provide new insights into the role of Vav1 in PDAC and, as targeting specific members of the Akt family is a promising therapeutic chance in solid tumors, they suggest that Vav1, by down-modulating Akt2, has potential as a molecular target in PDAC.

## 1. Introduction

Pancreatic ductal adenocarcinoma (PDAC) is one of the most malignant tumors, characterized by aggressive growth, which leads to a 5-year overall survival of less than 6%, without substantial progress in the last few years [1,2]. The increased activation of the PI3K/Akt pathway is common in PDAC and has been associated with higher histological tumor grade and worse prognosis [3,4,5]. In particular, both Akt1 and Akt2 are markedly activated in primary pancreatic tumors and tumor metastases, and Akt2 was positively correlated with resistance of PDAC to chemotherapy [6].

In cells from invasive breast tumors, we have recently demonstrated that activation and/or expression of specific Akt isoforms can be down-regulated by Vav1 [7], a multidomain protein physiologically expressed only in hematopoietic cells, in which it participates in cytoskeleton reorganization and gene transcription [8]. Vav1 is ectopically expressed in a number of non-hematopoietic cancers, including PDAC [9], in which this protein seems to be involved in signal transduction processes correlated with aggressive tumor phenotypes [10,11,12]. PDAC patients with Vav1-positive tumors have a worse prognosis compared to patients with Vav1-negative tumors [10,12], and the ability of Vav1 to promote the growth of pancreatic cancer cells was correlated to its best-known function as a guanosine exchange factor (GEF) [10]. As in breast tumors [13], Vav1 also shows a nuclear localization in PDAC [12], whose meaning is substantially different from that of cytoplasmic Vav1. In fact, as we demonstrated in breast tumors [13], PDAC patients with nuclear Vav1 in their primary cancer have a better prognosis than patients with only cytosolic Vav1 [12].

Considering that nuclear Vav1 is involved in gene transcription in myeloid leukemia cells [14,15] and that, in breast tumor cells, Vav1 plays a phenotype-related role in down-modulating the expression of specific Akt isoforms [7], the aim of this work was to establish whether Vav1 has a role in down-regulating the Akt pathway in pancreatic ductal adenocarcinoma. We found that the accumulation of Vav1 inside the nucleus inversely correlates with Akt2 expression in both PDAC-derived cells and pancreatic tumor tissues.

## 2. Experimental Section

All reagents were from Sigma (St. Louis, MO, USA) unless otherwise indicated.

### 2.1. Cell Lines and Tissue Samples

The pancreatic cancer cell lines HPAF-2, HPAC, and PL-45 were purchased from the American Type Culture Collection (Rockville, MD, USA). Cells were cultured in Dulbecco’s modified Eagle’s medium (DMEM, Gibco Laboratories, Grand Island, NY, USA) supplemented with 10% fetal bovine serum (FBS, Gibco Laboratories), 1% l-Glutamine, and 1% penicillin–streptomycin solution (Gibco Laboratories), at 37  °C in a humidified atmosphere of 5% CO_2_ in the air. Cells were periodically tested for mycoplasma and other contaminations.

Archived formalin-fixed, paraffin-embedded blocks from 59 patients diagnosed with primary pancreatic ductal adenocarcinoma were retrieved at the “Regina Elena National Cancer Institute” (Rome, Italy). Due to the retrospective nature of the study, performed on a cohort of cases without any patient-specific information, neither informed consent nor ethical approval was required.

### 2.2. Modulation of Vav1 Expression and Immunochemical Analysis

Silencing of Vav1 in HPAF-2 and HPAC cells and Vav1 over-expression in HPAF-2, HPAC, and PL-45 cell lines were performed with 1mg/mL Lipofectamine 2000 (Thermo Fisher Scientific, Waltham, MA, USA), essentially following previously reported procedures [7]. In particular, the down-modulation of Vav1 was performed by silencing protein with specific siRNAs (Santa Cruz Biotechnology, Santa Cruz, CA, USA) and Vav1 over-expression was obtained by transient transfection with a pEF plasmid expressing the human full-length Vav1. Non-silencing control siRNAs (Santa Cruz Biotechnology) and an empty vector were used as the control. To evaluate transfection efficiency, which resulted always higher than 70%, a non-silencing fluorescein-labeled duplex RNA (QIAGEN S.p.A., Milan, I) was used. The transfected cells were incubated at 37 °C in a 5% CO_2_ atmosphere for 48 h and then, subjected to evaluation of viability and to immunochemical, immunocytochemical, and real-time cell analysis.

To determine the number of viable cells, the Trypan Blue Exclusion Test was used, in which cells were suspended in PBS containing trypan blue and then, examined for their clear or blue cytoplasm with an inverted phase-contrast microscope (Diaphot, Nikon, Melville, NY, USA). For Western blot analysis, total lysates (50 μg protein) from cells under different experimental conditions were separated on 8.5% polyacrylamide denaturing gels and blotted into nitrocellulose membranes (GE Healthcare Life Science, Little Chalfont, UK). Membranes were reacted with antibodies directed against Vav1 (sc-8039)**,** p-Akt1/2/3 (sc-14032), Akt1 (sc-1618), and Akt2 (sc-5270), all purchased from Santa Cruz Biotechnology (Santa Cruz Biotechnology), and against β-tubulin, as previously reported [7]. Membranes were then incubated with peroxidase-conjugated secondary antibodies, and the immunocomplexes were detected by chemiluminescence using the ECL system (Perkin Elmer, Boston, MA, USA). The derived bands were acquired with an ImageQuantTM LAS 4000 biomolecular imager (GE Healthcare Life Science), and densitometrical analysis was performed with Image Quant TL software (GE Healthcare Life Science).

### 2.3. Real-Time Cell Assays of Migration and Invasion

Evaluation of cell migration and invasion was performed with the xCELLigence RTCA system (Real-Time Cell Analyzer System, Acea Biosciences Inc., San Diego, CA, USA), developed to monitor cell events in real-time, without the incorporation of dyes, by measuring electrical impedance, as previously reported [16]. For migration assays, 4 × 10^4^ cells/well were seeded onto the top chambers of CIM-16 plates with 8 μm pores (Roche Applied Science, Mannheim, D) and the bottom chambers were filled with medium containing 5% serum. The setup for analysis of invasiveness was the same as described for migration except that the upper side of the membranes was covered with a layer of Matrigel (BD Biosciences, San Josè, CA, USA) diluted 1:20 and the bottom chambers were filled with 10% serum containing medium. For both migration and invasion assays, the signal detection was programmed every 15 min for a total of 24 h. Impedance values were expressed as a dimensionless parameter (cell index, CI), where values were considered positive when >0.1.

### 2.4. Immunocytochemical and Confocal Analysis

Immunocytochemical analysis was performed by using a specific anti-Vav1 antibody (sc-132, Santa Cruz Biotechnology), as previously described [13]. Briefly, the different cell lines, grown onto glass slides, were fixed with 4% paraformaldehyde, washed in PBS, and reacted with the anti-Vav1 antibody in a Net Gel solution (150 mM NaCl, 5 mM EDTA, 50 mM Tris-HCl (pH 7.4), 0.05% NP40, 0.25% Carrageenan Lambda gelatin, and 0.02% Na azide) at room temperature. Samples were then labelled with a FITC-conjugated secondary antibody at room temperature in the dark. After washes with NET gel and PBS, samples were incubated with 0.5 μg/mL 4′,6-diamidino-2-phenylindole (DAPI), dried with ethanol, and mounted in glycerol containing 1,4-diazabicyclo [2.2.2] octane (DABCO) to retard fading. Fluorescent samples were analyzed with a Nikon Ci-L microscope (Nikon), and images were acquired with the NIS-ELEMENTS D software for a DS-Qi2Mc digital camera (Nikon).

For confocal analysis of Vav1 distribution, after labelling with the secondary antibody, samples were incubated with DRAQ5 Stain (Cell Signalling Technology, Leiden, N) for 30 min al 37 °C and mounted in glycerol containing DABCO. Images were obtained using a Zeiss LSM 50 laser scanning confocal microscope equipped with a 63× oil immersion Plan-Neofluar objective (Carl Zeiss, Göttingen, D). To measure Vav1 nuclear staining, digitized images were analyzed with ImageJ software (http://rsb.info.nih.gov/ij/), and fluorescence values were expressed as the mean of integrated density (IntDen) of at least 20 nuclei in three different areas.

### 2.5. Immunohistochemical Analysis

Immunohistochemical analysis was performed on tissue microarrays (TMA) constructed by removing 2 mm diameter cores of histologically confirmed ductal pancreatic cancer areas from 59 invasive primary tumors using a Manual Tissue Arrayer (MTA1, Beecher Instruments, Sun Prairie, WI, USA). Immunohistochemical staining of Vav1 on TMAs was performed with the anti-Vav1 antibody (sc-132, Santa Cruz Biotechnology) using the Anti-Rabbit HRP-DAB Cell and Tissue Staining Kit (R&D Systems, Minneapolis, MN, USA), according to the manufacturer’s instructions. The Vav1 staining of PDAC samples was validated with a second anti-Vav1 antibody (sc-7206, Santa Cruz Biotechnology) following the same procedure. Immunohistochemical analysis of Akt2 on TMAs was performed with an anti-Akt2 antibody (sc-5270, Santa Cruz Biotechnology) using the UltraVision LPValue Detection System—HRP Polymer (Ready-To-Use) (Thermo Fisher Scientific), as previously reported [13]. In particular, the slides were incubated in 3% (*v*/*v*) H_2_O_2_ to block endogenous peroxidases and the Ultra V Block reagent (Thermo Fisher Scientific) was used to reduce background. The arrays were then incubated at room temperature for 90 min with anti-Akt2 antibody in large volume UltrAbDiluent (Thermo Fisher Scientific). Staining was detected by the addition of a substrate/chromogen mix (DAB Quanto, Thermo Fisher Scientific) and the tissue microarrays sections were counterstained with Mayer’s hemalum solution (Bio-Optica, Milan, Italy). For all the immunohistochemical analyses, negative controls were obtained by omitting the primary antibody.

Stained tissue samples were analyzed by a Nikon Eclipse 90i (Nikon), and cell images were acquired with the NIS-ElementsF 2.30 software for a DS-2Mv digital camera (Nikon). For all samples, at least three different areas containing at least 100 cells were analyzed, and tumors were considered negative for Vav1 and/or Akt2 when less than 5% of cells were stained. In a scanned slide image, the staining intensity was estimated with ImageScope software (Aperio, Vista, CA, USA) as previously reported [13], and measured as Number of strong positive/Area (Nsp/µm^2^) ± SD. Nuclear staining of Vav1 in PDAC cells was expressed as fold changes relative to the cytoplasm, taken as 1 (i.e., relative Vav1 staining).

### 2.6. Statistical Analysis

Statistical analysis was performed by using the two-tailed, unpaired Student’s *t*-test. Statistics were computed using the GraphPad Prism 6.0 statistical package (GraphPad Software, San Diego, CA, USA), and *p* values < 0.05 were considered statistically significant.

## 3. Results and Discussion

Abnormal activation of the Akt pathway is the most common aberration of signal transduction in solid tumors, and considering its crucial role in regulating cell growth, proliferation, and survival [17], targeting Akt still constitutes an exciting objective for developing anticancer strategies [18,19]. Among the role played by the Akt isoforms in tumors, Akt1 is generally correlated with cell proliferation, survival, and apoptosis, while Akt2, which has become one of the most studied targets in diverse solid tumors, is mainly associated with cell migration and metastasis [20,21,22].

Pancreatic cancers are characterized by high levels of phosphorylated Akt that constitutes a significant prognostic indicator [4,5]. They also show high expression of Akt2 that result amplified in 10–20% and activated in up to 60% of PDAC [23], where it promotes cell growth and resistance to chemotherapy [6].

### 3.1. Vav1 Down-Modulates Motility and Akt2 Expression in PDAC-Derived Cell Lines

A peculiar modulation of the Akt family was recently shown in cells from invasive breast tumors with different phenotypes [13] and involved Vav1, a multidomain protein physiologically expressed in hematopoietic cells and whose ectopic expression in non-hematopoietic tissues was generally associated with the appearance of a tumor phenotype [24]. Despite its reported role as a negative prognostic molecule [25], in breast tumor cells, Vav1 down-modulates the activation status of Akt1, independently from tumor phenotypes, and reduces the expression of Akt1 in cells with an ER+ phenotype. Furthermore, the silencing of Vav1 in ER− cells induced the expression, but not the activation, of Akt2 [7].

**Figure 1:**
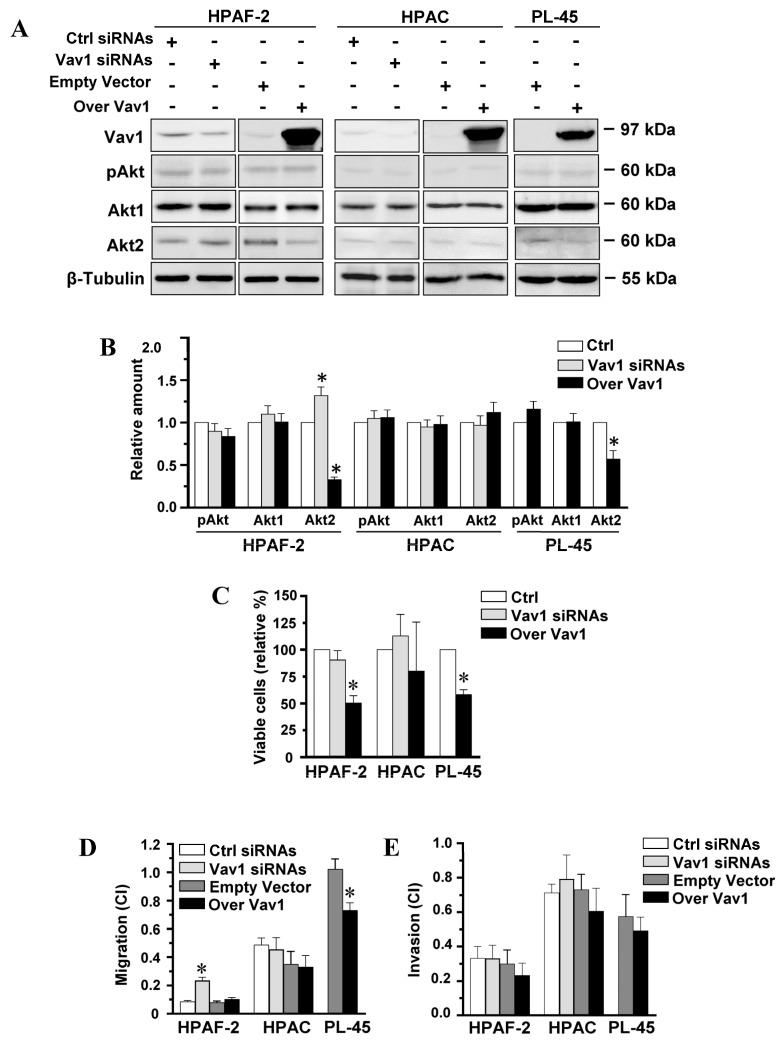
Vav1-dependent modulation of Akt2 and motility in PDAC-derived cell lines. (**A**) Representative Western blot analysis with the indicated antibodies of total lysates from HPAF-2, HPAC, and PL-45 pancreatic cancer cells transfected with siRNAs specific for Vav1 (Vav1 siRNAs) or with a construct expressing the full-length human Vav1 (Over Vav1). Scramble siRNAs (Ctrl siRNAs) and an empty vector were controls. In (**B**), levels of pAkt1/2/3, Akt1, and Akt2 as deduced from the densitometry of immunochemical bands normalized with β-Tubulin. Levels are shown as fold changes relative to the respective controls (Ctrl), taken as 1. In (**C**), percentage of viable cells relative to controls (Ctrl). In (**D**,**E**), xCELLigence-driven dynamic monitoring of migration and invasion of cells under the same experimental conditions. Cell Index (CI) after 24 h was reported. Error bars indicate ± SD from three experiments in triplicate. * *p* < 0.05.

Vav1 is also expressed in PDAC, where it was correlated with a worse prognosis [10,12]. To assess whether Vav1 may affect expression and/or activation of Akt in PDAC-derived cells with different malignant potential, the HPAF-2 and HPAC cell lines, expressing variable levels of Vav1, and PL-45 cells, negative for Vav1 (Figure 1A), were subjected to modulation of the Vav1 protein and evaluated for Akt expression and activation. Unlike breast tumor cells, immunochemical analysis with specific antibodies failed to reveal, in all PDAC cell lines, the effects of Vav1 on the whole Akt activation status (pAkt) and on the level of the most expressed Akt1 isozyme (Figure 1A). On the other hand, the expression level of Akt2 increased in HPAF-2, but not in HPAC cells, in which Vav1 was silenced and significantly decreased in HPAF-2 and PL-45, but not in HPAC, as a consequence of their transient transfection with a construct expressing the full-length human Vav1 (Figure 1A,B). The evaluation of the effects of the forced modulation of Vav1 on cell viability only showed a significant reduction in the number of HPAF-2 and PL45 living cells induced by the over-expression of the protein (Figure 1C). No effects on the viability of HPAC were revealed (Figure 1C), suggestive of a different involvement of Vav1 in the growth of PDAC-derived cells with different phenotypes.

As it is known that Akt2 is mainly associated with tumor cell migration and invasion [26], the PDAC-derived cells in which Vav1 was forcedly modulated were subjected to real-time cell assays of migration and invasion capability. As reported in Figure 1D, the basal motility of the examined cell lines inversely correlated with Vav1 expression, being the lowest in HPAF-2 cells, expressing the highest Vav1 levels, and the highest in the Vav1-negative PL-45. The forced modulation of Vav1 revealed that, in parallel to inducing the expression of Akt2, the silencing of Vav1 increased the migration rate of HPAF-2 (Figure 1D). On the other hand, no effects of the over-expression of Vav1 were observed on the very low basal motility of this cell line (Figure 1D). The high migration of PL-45 was counteracted by the over-expression of Vav1 (Figure 1D), which also induced a decrease in Akt2 in this cell line. No effects of the forced modulation of Vav1 were revealed in the migration rate of HPAC (Figure 1D), paralleling the absence of effects of silencing and over-expression of the protein on Akt2 levels and cell viability.

Neither the silencing nor the forced up-modulation of Vav1 was able to modify the invasion capability of the examined cell lines (Figure 1E). This suggests that Vav1 is involved in regulating the expression of Akt2 and, possibly, its enrollment in modulation of cell motility but indicates that other signaling events are necessary for the specific recruitment of this Akt isoform in invasion-related pathways.

Considering that, in pancreatic cancer, Akt2 is a crucial mediator in tumorigenesis and a potential target for developing new pancreatic cancer therapies [26], these data indicate that, in PDAC as in breast tumors, the role of Vav1 must be reconsidered based on its ability to down-modulate, to some extent, molecules known to be crucially involved in tumor signaling pathways.

### 3.2. Akt2 Expression Negatively Correlates with Nuclear Vav1 in PDAC-Derived Cell Lines and Tissues

The malignant role of Vav1 in solid tumors has generally been linked to its best known cytoplasmic function as a guanosine exchange factor for Rho/Rac GTPases [24], which ended to rearrange actin cytoskeleton, known to be at the basis of the motility of tumor cells [27]. However, additional and different roles of Vav1, correlated to its nuclear localization, were established in cells from acute myeloid leukemia (AML) [14]. In AML-derived cells, nuclear Vav1 is involved in gene transcription by cooperating with crucial transcription factors and with proteins variously involved in mRNA processing [15,28]. We have recently demonstrated that Vav1 accumulates inside the nucleus of breast tumor cells, and that high levels of nuclear Vav1 in primary breast tumors positively correlate with prognosis [13], indicating that the role of Vav1 in solid tumors needs to be investigated also considering its cellular distribution. As Vav1 was also described in the nucleus of some PDAC cells, and patients with nuclear Vav1 in their primary tumors showed a better prognosis than patients with only cytoplasmic Vav1 [12], the subcellular localization of this protein and its relationship with Akt2 was investigated in PDAC-derived cells and tissues.

**Figure 2:**
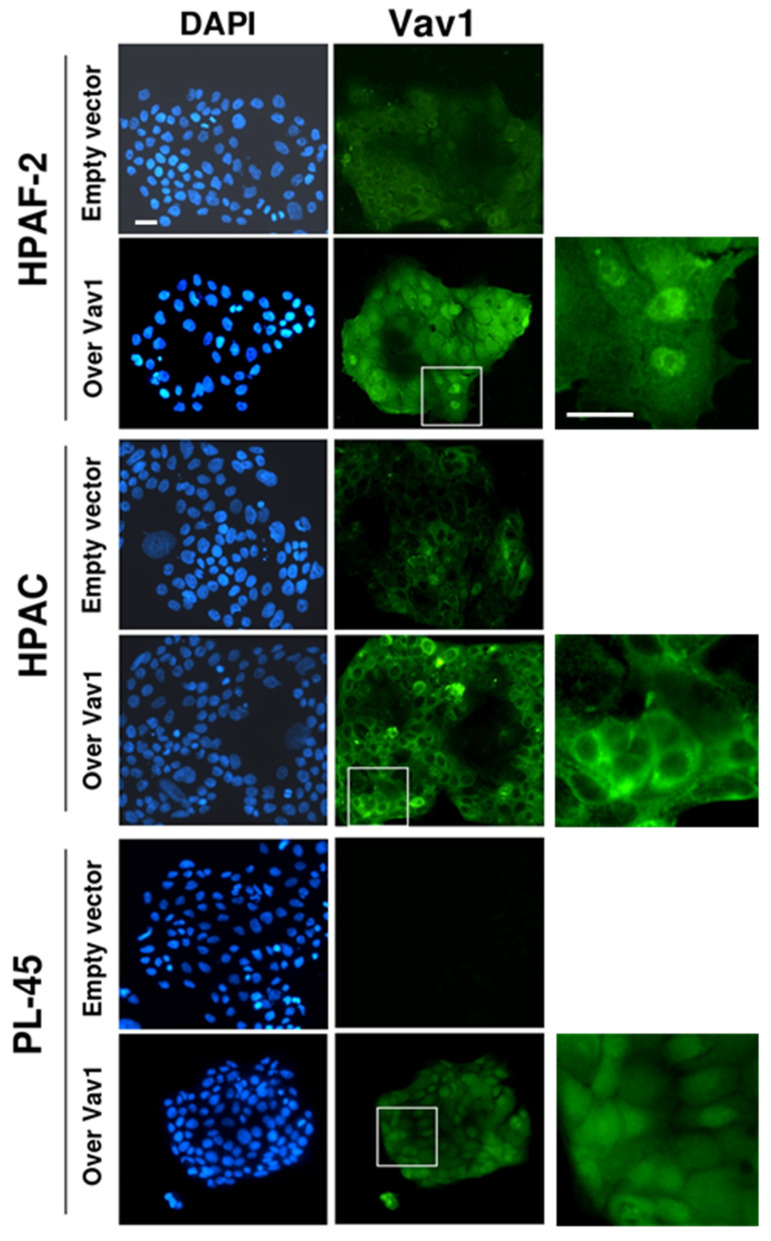
Subcellular localization of Vav1 in PDAC-derived cells. Representative fluorescence microscopy images of HPAF-2, HPAC, and PL-45 cells, in which cells were transiently transfected with a construct expressing the full-length human Vav1 (Over Vav1). Cells were subjected to immunocytochemical analysis with the anti-Vav1 antibody, and nuclei were counterstained with DAPI. On the right, zoomed images are shown. Bar: 20 µm.

**Figure 3:**
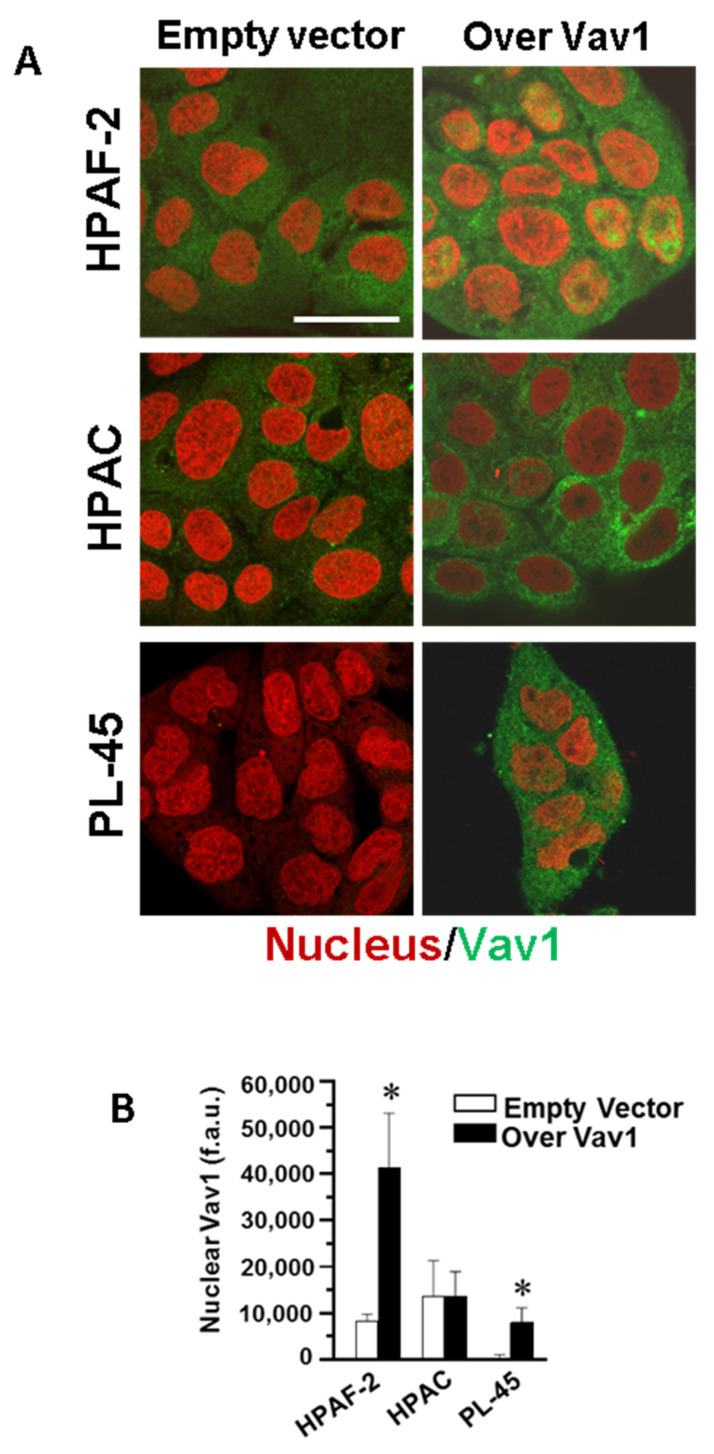
Confocal analysis of nuclear Vav1 in PDAC-derived cell lines. (**A**) Representative confocal images of HPAF-2, HPAC, and PL-45 cells transfected with a construct expressing the full-length human Vav1 (Over Vav1) and stained with the antibody against Vav1 (green). DRAQ5 stain was used to counterstain the nucleus (red). Bar = 20 μm. (**B**) Fluorescence intensity of nuclear Vav1 measured by the ImageJ software on digitized confocal images. F.a.u.: fluorescence arbitrary unit. Error bars indicate ± SD from a triplicate experiment. * *p* < 0.05.

By immunocytochemical analysis, we revealed that Vav1 is mainly localized in the cytoplasm of both the HPAF-2 and HPAC cell lines (Figure 2) and that, when over-expressed, Vav1 accumulated at the nuclear level in HPAF-2 and PL-45, while maintaining an almost exclusive cytoplasm localization in HPAC cells (Figure 2 and Figure 3). The mechanism/s at the basis of the different accumulation of Vav1 inside the nucleus of the examined cell lines may be correlated with the roles played by this protein in cytoplasm and/or nuclear compartments [8,14,15,28] and our results suggest that in HPAC cells, Vav1 acts predominantly at the cytoplasmic level, where it is retained.

Although our data do not clarify why over-expressed Vav1 failed to reach the nucleus of HPAC, they allow us to deduce that only when Vav1 accumulates in the nuclear compartment, there is a down-regulation of Akt2 expression. This is suggestive for a role of Vav1 in modulating gene transcription and/or mRNA processing in PDAC-derived cells, as we have already described for this protein in leukemic cells [15,28].

**Figure 4:**
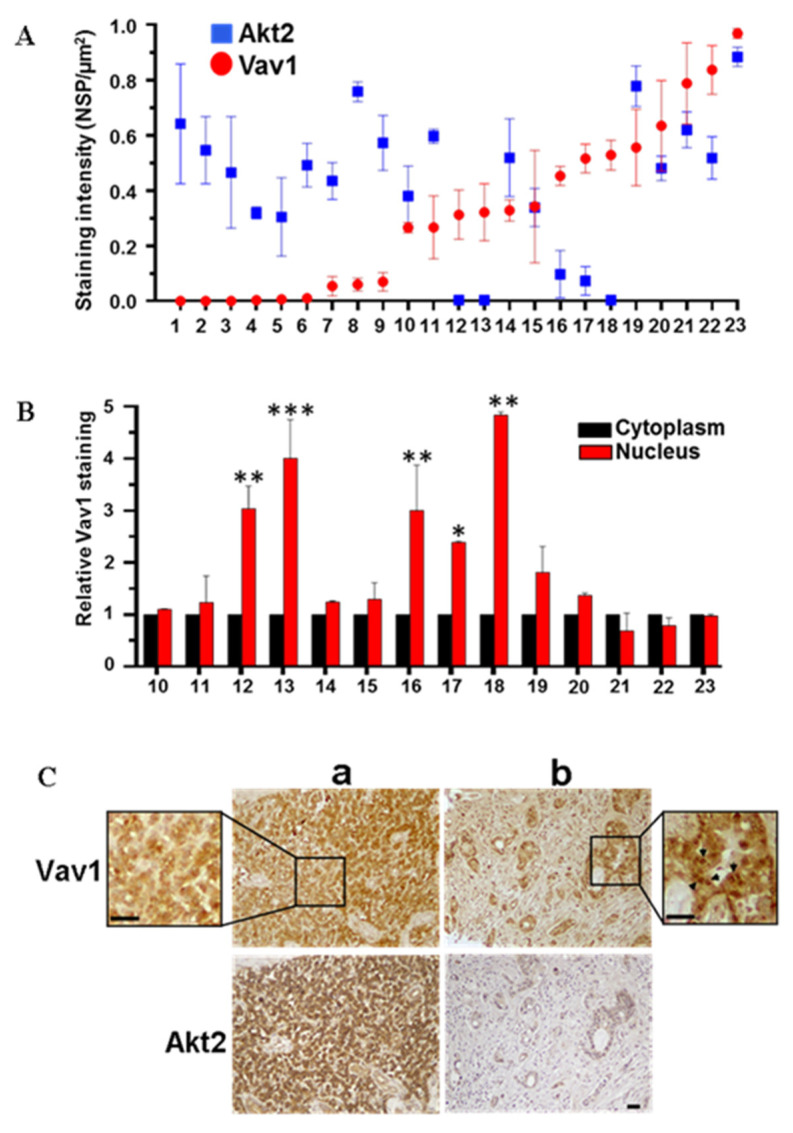
Expression and subcellular localization of Vav1 in pancreatic adenocarcinoma tissues. (**A**) Expression of Vav1 and Akt2 in PDAC samples. The staining intensity was expressed as Number of strong positive (Nsp) over area (µm^2^) ± SD. (**B**) Nuclear staining of Vav1 in PDAC samples. Values are shown as fold changes relative to the cytoplasm, taken as 1. * *p* ≤ 0.05; ** *p* ≤ 0.01; *** *p* ≤ 0.001. (**C**) Representative images of two human PDAC tissue sections after immunohistochemical analysis with anti-Vav1 and anti-Akt2 antibodies. Sample **a** represents a tumor in which Vav1 is uniformly distributed between cytoplasm and nucleus; sample **b** represents a sample in which Vav1 accumulates inside the nucleus. The arrows in the zoomed image indicate high nuclear Vav1 staining. Scale Bar = 50 μm.

The presence of Vav1 in the cell nucleus and its relationship with Akt2 was also investigated in primary tumors from PDAC patients. Of the 59 samples stained with the anti-Vav1 antibodies, 10 positive samples were excluded by the analysis due to the presence of a high number of white hematopoietic cells that intrinsically express high levels of Vav1. Of the evaluated samples, 18 over 49 (36.7%) were positive for Vav1 with both the used antibodies. Of the 59 samples analyzed, 21 (35.6%) were positive for Akt2, in line with the literature data [4,23]. Of the 47 samples evaluable for both Vav1 and Akt2, 24 were negative for both proteins, possibly reflecting early tumor stages. In 23 samples showing variable levels of Vav1 and/or Akt2, the Vav1/Akt2 relationship was examined, failing to reveal any apparent clear correlation between the amounts of the two proteins (Figure 4A). In fact, even if the majority of samples showed low Vav1 and high Akt2 or vice versa, suggesting an inverse correlation between proteins, a certain number of samples with high Vav1 showed high Akt2 expression (Figure 4A). The subcellular localization of Vav1 was then investigated, revealing the presence of the protein in both cytoplasm and nuclear compartments and its accumulation inside the nucleus in 5 over 14 samples (Figure 4B). Remarkably, only samples in which Vav1 accumulated inside the nucleus showed low Akt2 expression, while all samples in which Vav1 staining, although abundant, is uniformly distributed or mainly cytoplasmic, Akt2 levels were elevated (Figure 4A–C).

Despite the low number of analyzed samples, our data clearly indicate that when Vav1 is expressed and accumulates inside the nucleus of PDAC cells, the levels of Akt2 is down-modulated. This evidence allows us to correlate the results of a previous study on a large PDAC cohort, showing that patients with nuclear Vav1 in their primary tumors have a better prognosis than patients with only cytoplasmic Vav1 [12], with the reported negative prognostic role of Akt2 in pancreatic cancer [6]. In fact, we can believe that PDAC patients with nuclear Vav1 have a better prognosis also because nuclear Vav1 is involved in mechanism/s down-modulating the expression of Akt2.

## 4. Conclusions

As the disappointing prognosis of pancreatic ductal adenocarcinoma is mainly due to uncontrolled early spread of tumor cells, targeting molecules that specifically promote invasion and metastasis should be considered crucial for treating pancreatic cancer, even in the absence of detectable metastatic disease. Our results, demonstrating that Vav1 can down-modulate the levels of Akt2, whose role in PDAC malignancy is well-known, may be at the basis of further studies aimed to recognize Vav1, and particularly, nuclear Vav1, a new potential therapeutic target for some PDAC patients. Moreover, our data reveal the need for a reassessment of the role of Vav1 in pancreatic cancer and suggest that nuclear accumulation of this protein may be a PDAC prognostic factor.
